# Downregulation of Krüppel‐like factor 14 accelerated cellular senescence and aging

**DOI:** 10.1111/acel.13950

**Published:** 2023-08-08

**Authors:** Yuli Hou, Qiao Song, Yaqi Wang, Jing Liu, Yuting Cui, Xiaomin Zhang, Jingjing Zhang, Jingxuan Fu, Min Cao, Chi Zhang, Congcong Liu, Xiaoling Wang, Huanli Duan, Peichang Wang

**Affiliations:** ^1^ Department of Clinical Laboratory, Xuanwu Hospital, National Clinical Research Center for Geriatric Diseases Capital Medical University Beijing China; ^2^ Department of Clinical Laboratory Beijing Huairou Hospital Beijing China; ^3^ Departments of Pathology, Xuanwu Hospital Capital Medical University Beijing China

**Keywords:** aging, cellular senescence, KLF14, POLD1, transcriptional regulation

## Abstract

Aging has been considered as a risk factor in many diseases, thus, comprehensively understanding the cellular and molecular mechanisms of delayed aging is important. Here we investigated whether Krüppel‐like factor 14 (KLF14) is a suppressor of cellular senescence and aging. In our research, KLF14 levels significantly decreased not only in the lymphocytes of healthy people but also in the cells and tissues of mice with aging. We performed in vitro and in vivo experiments on cells and mice to reveal the function of KLF14 in aging. KLF14 deficiency facilitates cellular senescence and aging‐related pathologies in C57BL/6J mice, whereas KLF14 overexpression attenuates cellular senescence. Mechanistically, KLF14 delays aging by binding to the POLD1 promoter to positively regulate POLD1 expression. Remarkably, cellular senescence mediated by KLF14 downregulation could be alleviated by POLD1 expression. In addition, perhexiline, an agonist of KLF14, could delay cellular senescence and aging‐related pathologies in senescence‐accelerated P8 mice by inducing POLD1 expression, as perhexiline could enhance the effect of KLF14's transcription activation to POLD1 by elevating the binding level of KLF14 to the POLD1 promoter. Our data indicate that KLF14 might be a critical element in aging by upregulating POLD1 expression, indicating that the activation of KLF14 may delay aging and aging‐associated diseases.

## Graphical abstract

Yuli Hou et al. suggested Krüppel‐like factor 14 (KLF14), which is reduced with aging, as a crucial regulator of cellular senescence and aging. KLF14 delayed cellular senescence and aging‐related pathologies in mice by binding to the POLD1 promoter to positively regulate POLD1 expression. Activating KLF14‐mediated POLD1 expression using perhexiline could delay cellular senescence and aging‐related pathologies in senescence‐accelerated P8 mice.

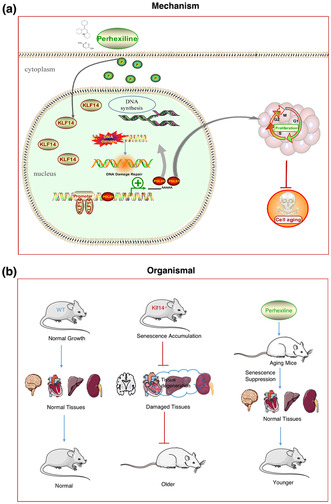

**Abbreviations**
ActD: actinomycin D
AD: Alzheimer's disease
CCK8: cell counting kit‐8 assay
ChIP: chromatin immunoprecipitation
DMSO: dimethyl sulfoxide
EdU: ethynyl deoxyuridine incorporation assay
GEO: Gene Expression Omnibus
H&E: hematoxylin and eosin
KLF14: Krüppel‐like factor 14
MDPL: mandibular hypoplasia, deafness, progeroid features, and lipodystrophy
MWM: Morris water maze
Pol δ: DNA polymerase δ
POLD1: DNA polymerase delta 1, catalytic subunit
RT‐qPCR: quantitative reverse transcription PCR
SAMP8: senescence‐accelerated P8
SAMR1: senescence‐resistant R1
SASP: senescence‐associated secretory phenotype
SA‐β‐Gal: senescence‐associated‐β‐galactosidase
TSS: transcription start site
WT: wild‐type

## 1 INTRODUCTION

Aging, which is characterized by a functional decline across multiple organ systems, is a major risk factor for a wide range of human diseases. Cumulative evidence has demonstrated that cellular senescence is a key hallmark of aging, which plays a crucial role in controlling aging and aging‐associated diseases (Song et al., 2020). Senescent cells are characterized by decreased cell proliferation, DNA synthesis, and repair capacity (Du et al., 2017), which were associated with the function of DNA polymerase δ (Pol δ).

Pol δ has a polymerase activity and 3′‐5′ exonuclease activity to participate in multiple DNA synthetic and repair processes (Robinson et al., 2021). Pol δ comprises four subunits, including three accessory subunits (p50/POLD2, p66/POLD3, and p12/POLD4) and a catalytic subunit (p125/POLD1) that provides the essential catalytic activities of the enzyme (Robinson et al., 2021). In particular, POLD1 has been found to play an important role on cellular senescence. The defective POLD1 function can induce cellular senescence and aging‐related pathologies, including the reduced lifespan of mice and the occurrence of mandibular hypoplasia, deafness, progeroid features, and lipodystrophy (MDPL), Werner syndrome, multiple tumors, and Alzheimer's disease (AD) (Lagisetty et al., 2022; Nicolas et al., 2016; Robinson et al., 2021). Thus, the regulation of POLD1 is important to understand the mechanism of aging.

Krüppel‐like factor 14 (KLF14), a member of the Krüppel‐like factor family, is a C2H2‐type zinc finger‐containing transcription factor (Chen et al., 2020). KLF14 is involved in the regulation of several cell functions, including cell proliferation, differentiation, and signal transduction, by regulating the expression of a wide range of target genes, such as FOXP3, ITGB1, NF‐κB, and WNT3A (Lyu et al., 2022; Sarmento et al., 2015; Weng et al., 2020). Recent studies have shown that KLF14 plays a protective role against multiple aging‐related diseases, including type 2 diabetes, atherosclerosis, and AD (Chen et al., 2020; Wezyk et al., 2018). Furthermore, decreased expression of KLF14 was associated with increased age in mice adipose tissue (Iwaya et al., 2018).

The POLD1 promoter is rich in GC dinucleotides, and it has many potential transcription factor binding sites (Nicolas et al., 2016). Based on previous reports, the transcription factor Sp1 could activate the POLD1 transcription by binding with its promoter (Antoniali et al., 2015). KLF14 binds similar GC‐rich DNA sequences with Sp1, which is preferred binding with the POLD1 promoter (Truty et al., 2009). However, the relationship between KLF14 and POLD1 is unknown in aging.

In this research, KLF14 was demonstrated to be a key transcriptional activator of POLD1, and the inhibition of the KLF14 expression could promote cellular senescence and aging‐related pathologies by reducing POLD1 expression. Our data suggested possibilities to modulate the KLF14‐POLD1 axis for delaying aging and preventing aging‐related diseases.

## 2 RESULTS

### 2.1 KLF14 expression decreased with aging, and the downregulation of KLF14 accelerated cellular senescence

**FIGURE 1:**
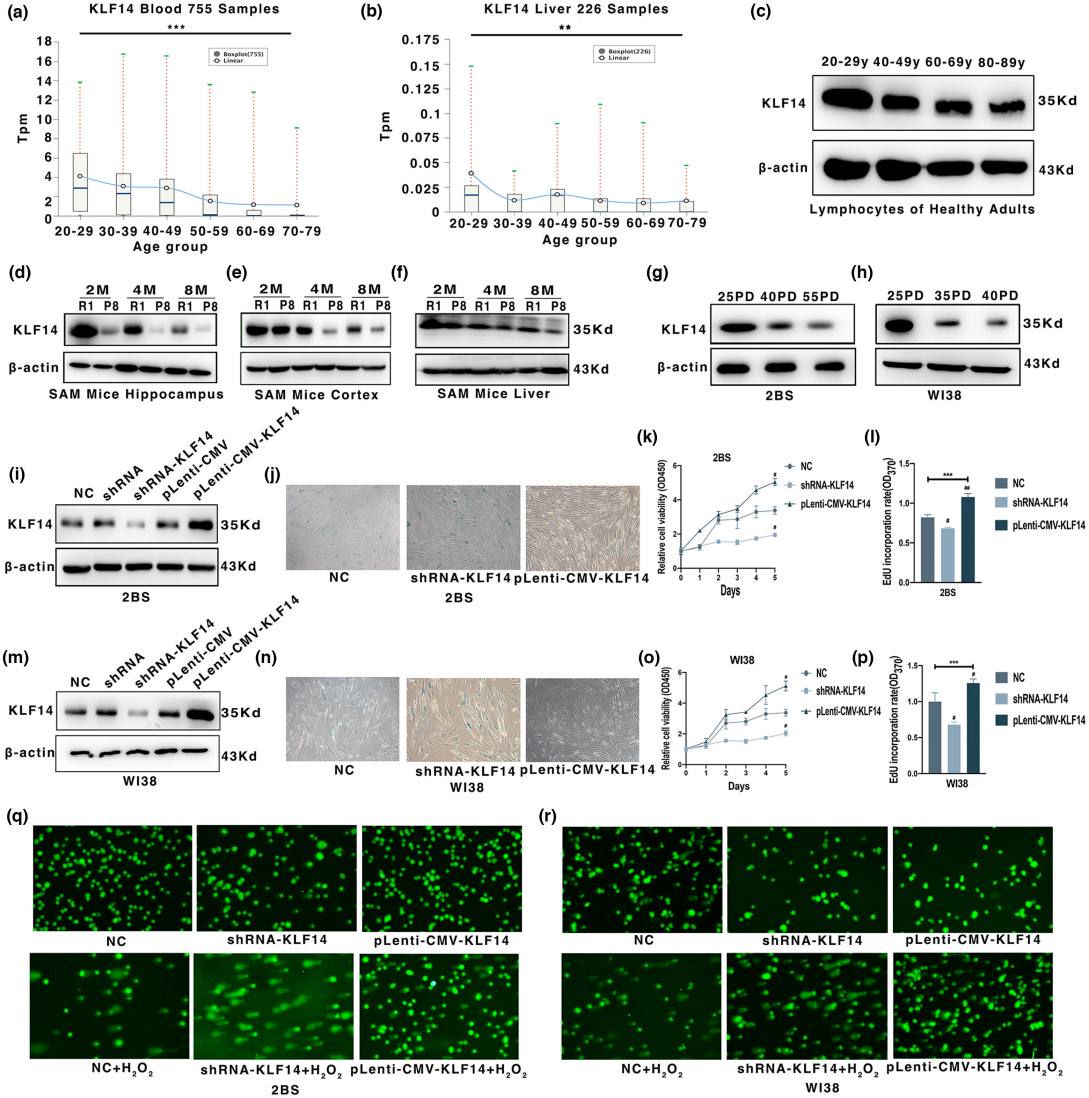
Krüppel‐like factor 14 (KLF14) expression decreased with aging, and the downregulation of KLF14 accelerated cellular senescence. (a) The average transcripts per million (TPM) of six age groups in 755 blood samples of healthy people is obtained from the ADEIP database. (b) The average TPM of six age groups in 226 liver samples of healthy people is obtained from the ADEIP database. (c) The protein expression of KLF14 in the lymphocytes of healthy people from different age groups. (d) The protein expression of KLF14 in the hippocampus of senescence‐resistant R1 (SAMR1) and senescence‐accelerated P8 (SAMP8) mice at the age of 2, 4, and 8 months. (e) The protein expression of KLF14 in the cortex of SAMR1 and SAMP8 mice at the age of 2, 4, and 8 months. (f) The protein expression of KLF14 in the liver of SAMR1 and SAMP8 mice at the age of 2, 4, and 8 months. (g) The protein expression of KLF14 in 2BS cells at the 25PD, 40PD, and 55PD. (h) The protein expression of KLF14 in WI38 cells at the 25PD, 35PD, and 40PD. (i) Knockdown and overexpression efficiency of KLF14 in 2BS cells at the protein level. (j) SA‐β‐gal staining of 2BS cells with KLF14 knockdown or overexpression. (k) Proliferative potential of 2BS cells with KLF14 knockdown or overexpression detected by CCK‐8 assay. (l) Ethynyl deoxyuridine (EdU) assay of 2BS cells with KLF14 knockdown or overexpression to detect the DNA synthesis ability. (m) Knockdown and overexpression efficiency of KLF14 in WI38 cells at the protein level. (n) SA‐β‐gal staining of WI38 cells with KLF14 knockdown or overexpression. (o) Proliferative potential of WI38 cells with KLF14 knockdown or overexpression detected by CCK‐8 assay. (p) EdU assay of WI38 cells with KLF14 knockdown or overexpression to detect the DNA synthesis ability. (q, r) DNA repair ability of 2BS cells and WI38 cells with KLF14 knockdown or overexpression detected by comet assay. Data were compared by one‐way ANOVA and Student's *t* test, and data were shown as mean ± SEM, with three independent experiments in each group. **p < 0.01, ***p < 0.005 by one‐way ANOVA test. ^#^ *p* < 0.05, ^##^ *p* < 0.01 by Student's *t* test (vs. NC).

In investigating the changes in KLF14 levels in various tissues with aging, we analyzed gene expression data obtained from the ADEIP database (http://gb.whu.edu.cn/ADEIP/), which integrates the age‐dependent gene expression and immune profiles across tissues (Liu et al., 2021). Intriguingly, KLF14 transcripts decreased in blood and liver tissues from different individuals with aging (Figure 1a,b). Consistent with the results in the ADEIP database, the levels of KLF14 in human peripheral blood lymphocytes (Figure 1c, Figure S1a,b) decreased with aging. Furthermore, senescence‐resistant R1 (SAMR1) mice and senescence‐accelerated P8 (SAMP8) mice, as well as human 2BS and WI38 cells, were selected to investigate the changes in KLF14 expression with aging. By immunoblotting analysis, the KLF14 protein level in the hippocampus (Figure 1d, Figure S1c), cortex (Figure 1e, Figure S1d), and liver (Figure 1f, Figure S1e) tissues of SAMR1 and SAMP8 mice decreased with aging. Subsequently, levels of KLF14 in senescent 2BS or WI38 cells decreased significantly compared with those in young 2BS or WI38 cells (Figure 1g,h, Figure S1f–i).

In verifying the functional importance of KLF14 in cellular senescence, knockdown and overexpression methods were used to regulate KLF14 expression in 35 population doubling (PD) 2BS and WI38 cells. The results showed that the expression of KLF14 was dramatically downregulated after transfection with shRNA‐KLF14 and upregulated with pLenti‐CMV‐KLF14 transfection in 2BS and WI38 cells (Figure 1i,m, Figure S1j–m). Cellular senescence is characterized by elevated levels of endogenous beta‐gal activity at pH 6.0, which may be identified by SA‐β‐gal assay (Jiang et al., 2022). The results showed that cells with decreased expression level of KLF14 displayed a higher frequency of SA‐β‐gal staining. Meanwhile, a lower frequency of SA‐β‐gal staining was found in cells with increased expression of KLF14 (Figure 1j,n). Senescence phenotypes, including low cell viability detected by CCK8 assay, low DNA synthesis rate, and low DNA repair ability when treated with 100 μM H_2_O_2_, were observed in cells transfected with shRNA‐KLF14 (Figure 1k,l,o–r). Collectively, these data indicated that KLF14 expression decreased with aging, and KLF14 suppressed cellular senescence by facilitating cell proliferation, DNA synthesis, and DNA repair ability.

### 2.2 KLF14 bound to the POLD1 promoter to activate POLD1 transcription

**FIGURE 2:**
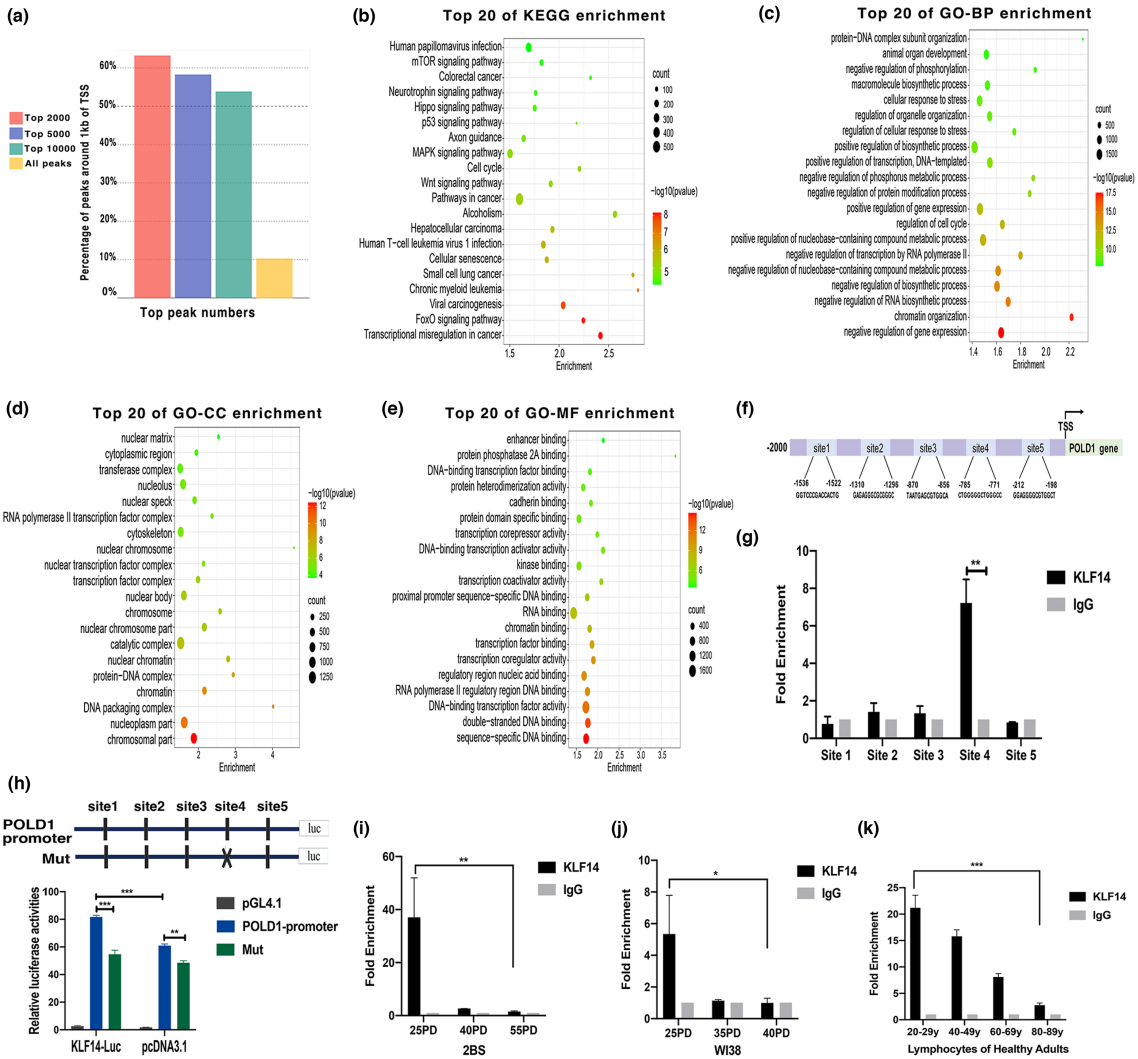
Krüppel‐like factor 14 (KLF14) bound to POLD1 promoter to activate POLD1 transcription. (a) The binding peak distribution of KLF14 ChIP‐seq data analysis (GSM3635904) by using Cistrome‐GO. (b) The top 20 KEGG enrichment terms of pathway analysis of the genes that are predicted to bind with the KLF14 transcription factor. (c) The top 20 functional terms of GO‐BP enrichment analysis of the genes that are predicted to bind with the KLF14 transcription factor. (d) The top 20 functional terms of GO‐CC enrichment analysis of the genes that are predicted to bind with the KLF14 transcription factor. (e) The top 20 functional terms of GO–MF enrichment analysis of the genes that are predicted to bind with the KLF14 transcription factor. (f) Putative KLF14 binding sites on human POLD1 promoter predicted by the JASPAR database. (g) The binding sites of KLF14 to the POLD1 promoter were analyzed by ChIP‐qPCR assay. (h) Promoter activities were determined by luciferase assay in HEK‐293 cells transfected with KLF14 or pcDNA3.1 and human POLD1 promoter or mutation luciferase reporter plasmid (−2000 to transcription start site). (i) Binding level of KLF14 at the POLD1 promoter in 2BS cells from different population doublings (PDs). (j) Binding level of KLF14 at the POLD1 promoter in WI38 cells from different PDs. (k) Binding level of KLF14 at the POLD1 promoter in lymphocytes from healthy people of different ages. Data were shown as mean ± SEM of three separate experiments (*p < 0.05, **p < 0.01, ***p < 0.001). Statistical analyses were performed using *t* test (two groups) or one‐way ANOVA (more than two groups) in Prism.

Krüppel‐like factor 14 is a key transcription factor, and it can regulate its target gene transcription by binding to DNA‐specific sequences to participate in a diverse array of cellular processes (Chen et al., 2020). In obtaining a comprehensive insight into the molecular mechanism by which KLF14 regulates cellular senescence, chromatin immunoprecipitation sequencing (ChIP‐seq) data (GSM3635904) downloaded from the Gene Expression Omnibus (GEO) database was used to explore the potential KLF14 binding sites and functional enrichment of target genes by using Cistrome‐GO (http://go.cistrome.org/) (Zheng et al., 2019). First, the results revealed that the KLF14 binding peaks most at 1 kb of the gene transcription start site (TSS), which belongs to the region of the gene promoter, indicating that KLF14 may bind to the target gene promoter to display its function (Figure 2a). Subsequently, functional enrichment showed that the targets of KLF14 were primarily bound in cellular senescence and cell differentiation‐related pathways (Figure 2b). The top 20 terms of GO enrichment analyses of KLF14 targets are illustrated in Figure 2c–e. GO‐BP function enrichment analysis showed that the target genes of KLF14 were significantly enriched in the cellular response to stress, regulation of cell cycle, and chromatin organization, which all play an important part in senescence (Sławińska & Krupa, 2021) (Figure 2c). Of the GO‐CC terms, chromatin, nuclear matrix, and protein–DNA complex were primarily enriched in targets of KLF14 (Figure 2d). GO–MF function enrichment analysis showed that the target genes of KLF14 were significantly enriched in sequence‐specific DNA binding, double‐stranded DNA binding, DNA‐binding transcription factor activity, and so on, which are consistent with the function of the transcription factor (Figure 2e).

We identified the key transcriptional target gene in the context of cellular senescence to investigate the mechanism by which the transcription factor KLF14 restricts cellular senescence. Notably, POLD1, as a marker of senescence to regulate cellular function, was identified to be bound with KLF14 (Song et al., 2022). Five putative sequences in the POLD1 promoter were found to interact with KLF14 using computational analyses on the JASPAR database. The five binding sites were located within −1536 to −1522 (site 1), −1310 to −1296 (site 2), −870 to −856 (site 3), −785 to −771 (site 4), and −212 to −198 (site 5, Figure 2f). In confirming this observation, we performed ChIP‐qPCR assay with anti‐KLF14 and anti‐IgG antibodies, and the result revealed that KLF14 binds primarily to the Site 4 region of the POLD1 promoter (Figure 2g). In determining the function of KLF14‐binding to the POLD1 promoter in the transcription of POLD1 gene, we generated luciferase reporter constructs of human POLD1 promoter with the overall length and mutant KLF14‐binding site. Each of these fragments and the control reporter (basic pGL4.1 empty vector) were co‐transfected with KLF14 or the control vector (pcDNA3.1) in HEK‐293 cells, and the luciferase activity was detected. The POLD1 promoter with the overall length showed increased luciferase reporter activity with KLF14 overexpression compared with the control vector, indicating that KLF14 could promote the activity of the POLD1 promoter. However, KLF14 could hardly activate POLD1 promoter transcription when Site 4 of the POLD1 promoter was mutated (Figure 2h). This evidence indicated that KLF14 promotes POLD1 transcription by binding primarily to Site 4 of the POLD1 promoter.

Furthermore, the ChIP assay was conducted by using 2BS cells, WI38 cells of different PDs, and lymphocyte cells from different ages of healthy people to investigate whether the binding level of KLF14 with the POLD1 promoter changed with aging. Our results showed that the KLF14‐binding level gradually decreased in 2BS, WI38, and lymphocyte cells with aging (Figure 2i–k). These results indicated that the KLF14‐binding level of the POLD1 promoter is dynamically regulated by aging, and KLF14 is a crucial transcription factor controlling POLD1 transcription in aging.

### 2.3 POLD1 expression was downregulated with aging and positively regulated by KLF14

**FIGURE 3:**
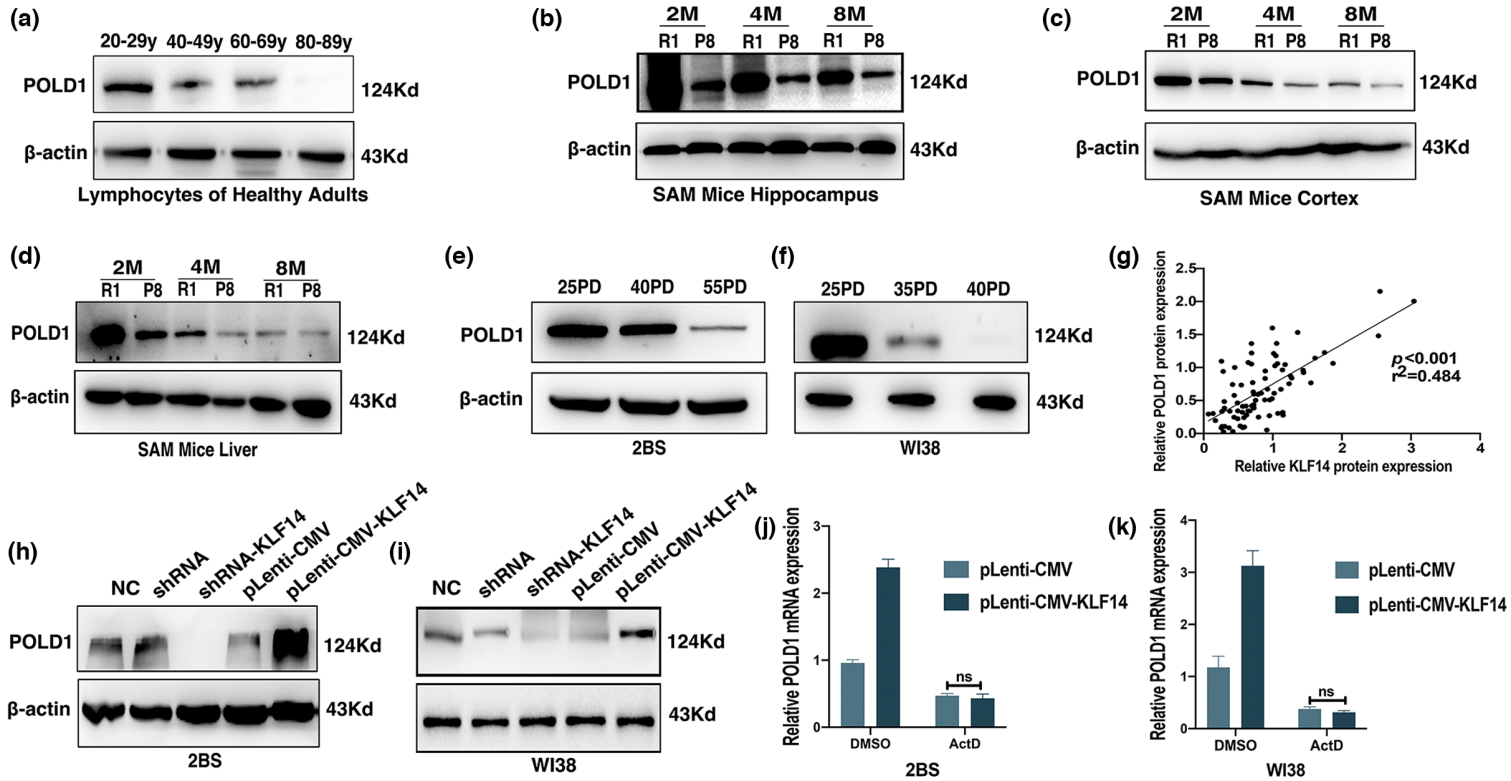
POLD1 expression was downregulated with aging and positively regulated by Krüppel‐like factor 14 (KLF14). (a) The protein level of POLD1 in the lymphocytes of healthy people of different ages. (b) The POLD1 protein expression in the hippocampus of senescence‐resistant R1 (SAMR1) and senescence‐accelerated P8 (SAMP8) mice at different ages in months. (c) Protein expression levels of POLD1 in the cortex of SAMR1 and SAMP8 mice at different ages in months. (d) Protein expression levels of POLD1 in the liver of SAMR1 and SAMP8 mice at different ages in months. (e) Protein expression levels of POLD1 in different population doublings (PDs) of 2BS cells. (f) Protein expression levels of POLD1 in different PDs of WI38 cells. (g) The relationship between the protein levels of KLF14 (*X*‐axis) and POLD1 (*Y*‐axis) in lymphocytes of healthy adults, mice, and cells. (h, i) Representative western blots of the protein expression level of POLD1 in 2BS (h) and WI38 (i) cells transfected with shRNA‐KLF14, pLenti‐CMV‐KLF14, or the control vector. (j, k) mRNA level of POLD1 in 2BS (j) and WI38 (k) cells transfected with pLenti‐CMV‐KLF14 or the control vector and treated with actinomycin D (10 μg/mL) or DMSO. Data were presented as mean ± SEM of three separate experiments (ns *p* > 0.05). Statistical analyses were performed using one‐way ANOVA, Student's *t* test, and linear regression analysis in Prism.

The lymphocytes from healthy people of different ages (20–29, 40–49, 60–69, and 80–89 years), tissues of the hippocampus, cortex, and liver from SAMR1 and SAMP8 mice at different ages in months (2, 4, and 8 months), 2BS (25, 40, and 55 PDs) and WI38 (25, 35, and 40 PDs) cells were used to detect POLD1 expression. The POLD1 level in human lymphocytes of old healthy people (80–89 years old) was lowest among all the age groups, which is consistent with the expression of KLF14 (Figure 3a, Figure S2a,b). Furthermore, the progressive decrease of POLD1 expression with aging was found not only in the tissues of senescence‐accelerated mice (Figure 3b–d, Figure S2c–e) but also in 2BS (Figure 3e, Figure S2f,g) and WI38 (Figure 3f, Figure S2h,i) cells, which is consistent with the literature (Gao et al., 2019). Moreover, we found that protein and mRNA levels of POLD1 were positively correlated with KLF14 expression levels in aging (protein level, *p* < 0.001, *r* ^2^ = 0.484; mRNA level, *p* < 0.05, *r* ^2^ = 0.294; Figure 3g, Figure S2j). These data also indicated that KLF14 was closely associated with POLD1 expression in aging.

Based on the positive relationship between KLF14 and POLD1 expression in aging and the results of KLF14 binding to the POLD1 promoter to activate POLD1 transcription, we explored the effect of KLF14 on POLD1 expression during cellular senescence. Thus, we performed shRNA‐KLF14 and pLenti‐CMV‐KLF14 to knockdown or overexpress KLF14 genes in 2BS cells and WI38 cells. The transfection efficiency of KLF14 is shown in Figure 1i,m. We found that the mRNA and protein expression of POLD1was downregulated in 2BS and WI38 cells with shRNA‐KLF14 transfection and upregulated in cells with pLenti‐CMV‐KLF14 transfection (Figure 3h,i, Figure S2k–n). These results demonstrated that KLF14 positively regulates POLD1 expression.

In addition, actinomycin D (ActD), a transcriptional inhibitor (Guo et al., 2015), was used in 2BS and WI38 cells transfected with pLenti‐CMV‐KLF14. Dimethyl sulfoxide (DMSO) was used as the negative control. The results showed that POLD1 upregulation induced by KLF14 overexpression was blocked by ActD (10 μg/mL; Figure 3j,k, Figure S2o,p). These data also verified that KLF14 regulates POLD1 gene expression through transcription.

### 2.4 Silencing POLD1 reversed rejuvenation induced by KLF14 overexpression

**FIGURE 4:**
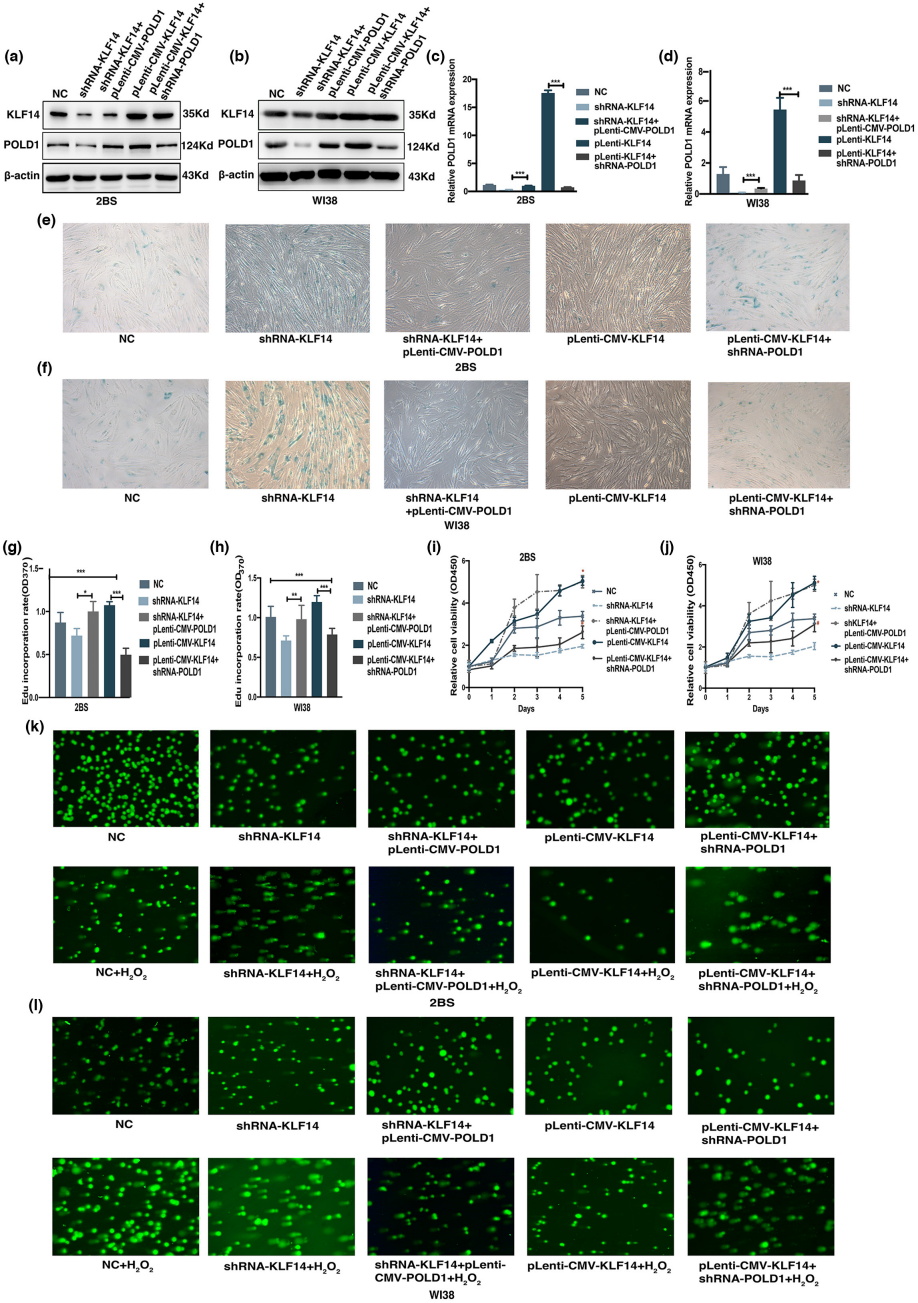
Silencing POLD1 reversed rejuvenation induced by Krüppel‐like factor 14 (KLF14) overexpression. (a, b) Representative western blots of KLF14 and POLD1 proteins in 2BS (a) and WI38 (b) cells co‐transfected with KLF14 and POLD1 lentivirus. (c, d) The mRNA expression of POLD1 in 2BS (c) and WI38 (d) cells co‐transfected with KLF14 and POLD1 lentivirus. (e, f) SA‐β‐gal staining of 2BS (e) and WI38 (f) cells co‐transfected with KLF14 and POLD1 lentivirus. (g, h) Ethynyl deoxyuridine assay of 2BS (g) and WI38 (h) cells co‐transfected with KLF14 and POLD1 lentivirus. (i, j) Proliferative potential of 2BS (i) and WI38 (j) cells co‐transfected with KLF14 and POLD1 lentivirus detected by CCK‐8 assay. (k, l) DNA repair ability of 2BS (k) and WI38 (l) cells co‐transfected with KLF14 and POLD1 lentivirus detected by comet assay. Data were compared by one‐way ANOVA and Student's *t* test, and data were shown as mean ± SEM, with three independent experiments in each group. *p < 0.05, **p < 0.01, ***p < 0.005. ^#^ *p* < 0.05 by Student's *t* test (shRNA‐KLF14+pLenti‐CMV‐POLD1 vs. shRNA‐KLF14, pLenti‐CMV‐KLF14+shRNA‐POLD1 vs. pLenti‐CMV‐KLF14).

Given that KLF14 could regulate the expression of POLD1 and the progression of cellular senescence, we tested whether the knockdown or overexpression of POLD1 could interfere with the effect of KLF14 on cellular senescence. Thus, we performed rescue experiments in 2BS and WI38 cells. We overexpressed POLD1 in cells with KLF14 knockdown (shRNA‐KLF14+pLenti‐CMV‐POLD1) and POLD1 knockdown in cells with KLF14 overexpression (pLenti‐CMV‐KLF14+shRNA‐POLD1). The cells were divided into five groups: NC, shRNA‐KLF14, shRNA‐KLF14+pLenti‐CMV‐POLD1, pLenti‐CMV‐KLF14, and pLenti‐CMV‐KLF14+shRNA‐POLD1. The results showed that the expression level of POLD1 was higher in cells co‐transfected with pLenti‐CMV‐POLD1 and shRNA‐KLF14 than in cells transfected solely with shRNA‐KLF14. Meanwhile, the increased expression of POLD1 induced by KLF14‐overexpression was reversed after transfection with the shRNA‐POLD1 lentivirus (Figure 4a–d, Figure S3a–f).

Furthermore, the rejuvenation of the cells induced by KLF14 overexpression could be reversed by POLD1 downregulation. The decreased SA‐β‐gal activity in cells solely transfected with pLenti‐CMV‐KLF14 was significantly alleviated by POLD1 silencing (Figure 4e,f). The increased DNA synthesis and cell proliferation ability in cells solely transfected with pLenti‐CMV‐KLF14 were significantly attenuated by POLD1 silencing (Figure 4g–j). A similar result was also presented in the comet assay, and the DNA damage repair ability in cells co‐transfected with shRNA‐POLD1 and pLenti‐CMV‐KLF14 was significantly lower than that transfected only with pLenti‐CMV‐KLF14 (Figure 4k,l). In addition, the senescence phenotype in 2BS and WI38 cells transfected with shRNA‐KLF14 was rescued by POLD1 overexpression (Figure 4e–l). These results demonstrated that POLD1 is the primary factor mediating the function of KLF14 in cellular senescence.

### 2.5 KLF14 deficiency promoted mouse aging

**FIGURE 5:**
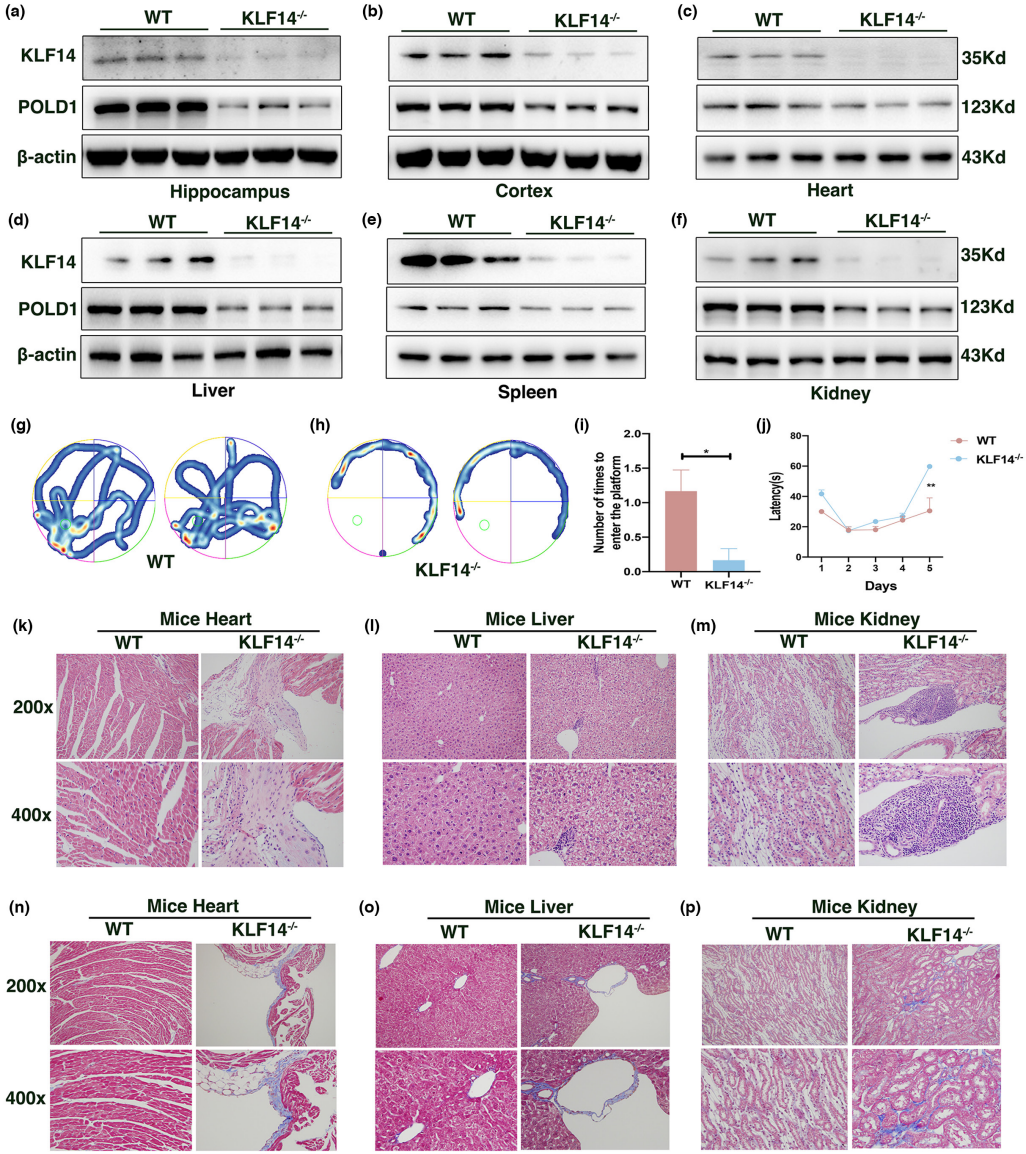
Krüppel‐like factor 14 (KLF14) deficiency promoted mouse aging. (a–f) KLF14 and POLD1 proteins in the hippocampus (a), cortex (b), heart (c), liver (d), spleen (e), and kidney (f) of 6‐month‐old wild‐type (WT) and KLF14^−/−^ mice (n = 6 per group) were determined by western blot. (g, h) The Morris water maze (MWM) test representative trajectories of WT (g) and KLF14^−/−^ (h) mice in the probe trial. (i) The number of times the 6‐month‐old WT and KLF14^−/−^ mice (n = 6 per group) entered the platform during the MWM test. (j) Latency(s) to find the platform of 6‐month‐old WT and KLF14^−/−^ mice (n = 6 per group) during the MWM test. (k–m) Representative H&E staining images of the heart (k), liver (l), and kidney (m) of 6‐month‐old WT and KLF14^−/−^ mice. (n–p) Representative Masson trichrome staining images of the heart (n), liver (o), and kidney (p) in 6‐month‐old WT and KLF14^−/−^ mice. Data were compared by Student's *t* test, and data were shown as mean ± SEM (*p < 0.05, **p < 0.01).

For the in vivo assay to determine the role of KLF14 in aging, we generated KLF14 knockout (KLF14^−/−^) mice. Quantitative reverse transcription PCR (RT‐qPCR) and western blot analysis confirmed a decreased expression of KLF14 in the hippocampus, cortex, heart, liver, spleen, and kidney tissues of KLF14^−/−^ mice, indicating that the gene has been knocked down (Figure 5a–f, Figure S4a–l). Meanwhile, we observed a significant downregulation of POLD1 in tissues from KLF14^−/−^ mice compared with wild‐type (WT) negative control mice, underscoring the role of KLF14 in the regulation of POLD1 expression (Figure 5a–f, Figure S4a–l).

Considering that cognitive decline commonly occurs with aging and as an early marker of pathological aging (Zlatar et al., 2022), the cognitive function was performed using the Morris water test to assess the aging status in mice. In the probe trial test, the number of platform area crossings in KLF14^−/−^ mice was less than that in the WT group (Figure 5g–i). The group of KLF14^−/−^ mice showed a significant increase in the latency time when compared with the WT group (Figure 5j). Collectively, these results showed a significant impairment of cognitive function in KLF14^−/−^ mice.

Moreover, hair loss occurred in 3‐, 6‐, and 11‐month‐old KLF14^−/−^ mice, along with a significant reduction in the overall size of the spleen in 6‐month‐old KLF14^−/−^ mice (Figure S4m–p,t–w). Afterward, tissues were examined with hematoxylin and eosin (H&E) staining to evaluate the nature and severity of lesions. Cardiac hypertrophy in the heart, inflammatory infiltration, and vacuolation in the liver, inflammatory infiltration, and glomerular sclerosis in the kidney were also observed in 6‐ and 11‐month‐old KLF14 knockdown mice (Figure 5k–m, Figure S4q–s,x–z). Aged tissue is characterized by increased fibrosis, which leads to aging‐associated diseases (Ma et al., 2019). Using Masson's trichrome staining to identify fibrotic tissue, we showed clear evidence of fibrosis in the heart, liver, and kidney of 3‐, 6‐, and 11‐month‐old KLF14^−/−^ mice (Figure 5n–p, Figure S4a1–c1). Collectively, the findings demonstrated that KLF14 deficiency decreases POLD1 expression and promotes aging‐related pathologies, thereby promoting premature aging in mice.

### 2.6 Perhexiline delayed cellular senescence by activating KLF14

**FIGURE 6:**
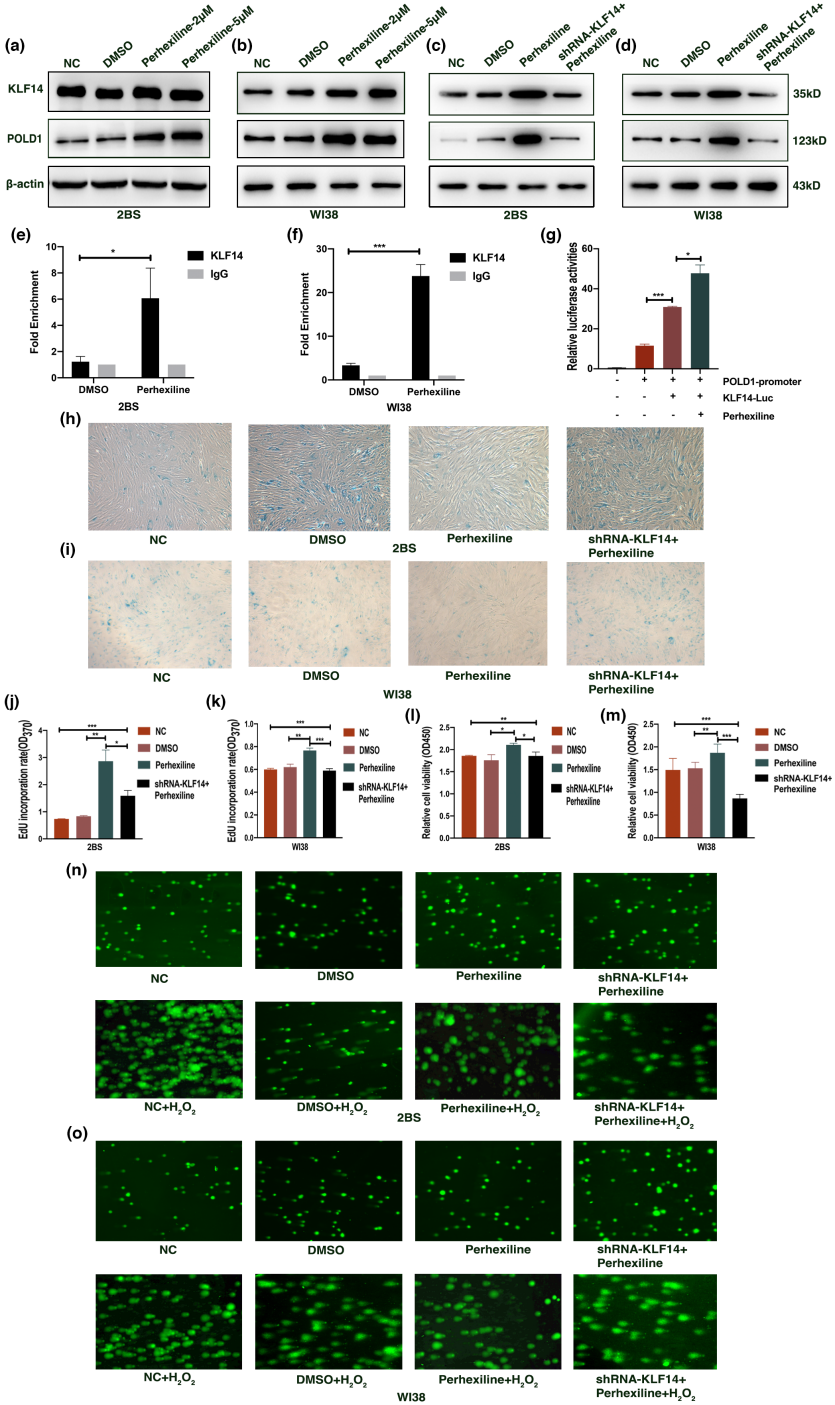
Perhexiline delayed cellular senescence through activating Krüppel‐like factor 14 (KLF14). (a, b) Representative western blots of the protein expression levels of KLF14 and POLD1 in 2BS (a) and WI38 (b) cells treated with DMSO or perhexiline. (c, d) Representative western blots of the protein expression levels of KLF14 and POLD1 in 2BS (c) and WI38 (d) cells, which were transfected with shRNA‐KLF14 or the control vector incubated with 5 μM of perhexiline. (e, f) ChIP‐qPCR assay of 2BS (e) and WI38 (f) cells treated with 5 μM of perhexiline or DMSO. (g) Luciferase activity of reporters was analyzed in HEK‐293 cells transfected with KLF14 or pcDNA3.1 and human POLD1 promoter luciferase reporter plasmid after 24 h of treatment with 5 μM of perhexiline or DMSO. (h, i) SA‐β‐gal staining of 2BS (h) and WI38 (i) cells which were transfected with shRNA‐KLF14 or the control vector incubated with 5 μM of perhexiline. (j, k) Ethynyl deoxyuridine assay of 2BS (j) and WI38 (k) cells which were transfected with shRNA‐KLF14 or the control vector incubated with 5 μM of perhexiline. (l, m) Proliferative potential of 2BS (l) and WI38 (m) cells which were transfected with shRNA‐KLF14 or the control vector incubated with 5 μM of perhexiline detected by CCK‐8 assay. (n, o) DNA repair ability of 2BS (n) and WI38 (o) cells which were transfected with shRNA‐KLF14 or the control vector incubated with 5 μM of perhexiline detected by comet assay. Data were compared by one‐way ANOVA and Student's *t* test, and data were shown as mean ± SEM, with three independent experiments in each group (*p < 0.05, **p < 0.01, ***p < 0.005).

Based on the above mentioned observations, we assumed whether perhexiline, as an agonist of KLF14, could inhibit cellular senescence. DMSO was used as a negative control. Consistent with a previous study (Guo et al., 2015), the increased expression of KLF14 was observed in cells cultured with perhexiline (Figure 6a,b, Figure S5a–d). Similar to KLF14 overexpression, a significant increase of POLD1 expression was also detected in cells cultured with perhexiline (Figure 6a,b, Figure S5a–d), but this effect was largely abolished in KLF14 knockdown cells (Figure 6c,d, Figure S5e–h), indicating that the perhexiline‐associated increase of POLD1 might depend on KLF14. In our mechanistic experiments, we observed that perhexiline elevates the binding level of KLF14 to the POLD1 promoter. In particular, perhexiline could also enhance the effect of KLF14's transcription activation on POLD1 (Figure 6e–g).

In examining the potential role of perhexiline on cellular senescence, we detected the senescence characteristics of cells treated with perhexiline. As shown in Figure 6h,i, we observed a decreased activity of SA‐β‐Gal in cells treated with perhexiline (5 μM), and this effect was attenuated in KLF14 knockdown cells. In addition, cell proliferation, DNA synthesis, and DNA damage repair ability were detected in cells treated with perhexiline. Our results showed that cells treated with perhexiline had a higher cell proliferation, DNA synthesis ability, and DNA damage repair ability compared with the cells treated with DMSO. In particular, perhexiline‐attenuated cellular senescence was diminished in cells transfected with shRNA‐KLF14 (Figure 6j–o). These results indicated that perhexiline inhibited cellular senescence by activating KLF14.

### 2.7 Perhexiline rescued aging phenotypes in SAMP8 mice

**FIGURE 7:**
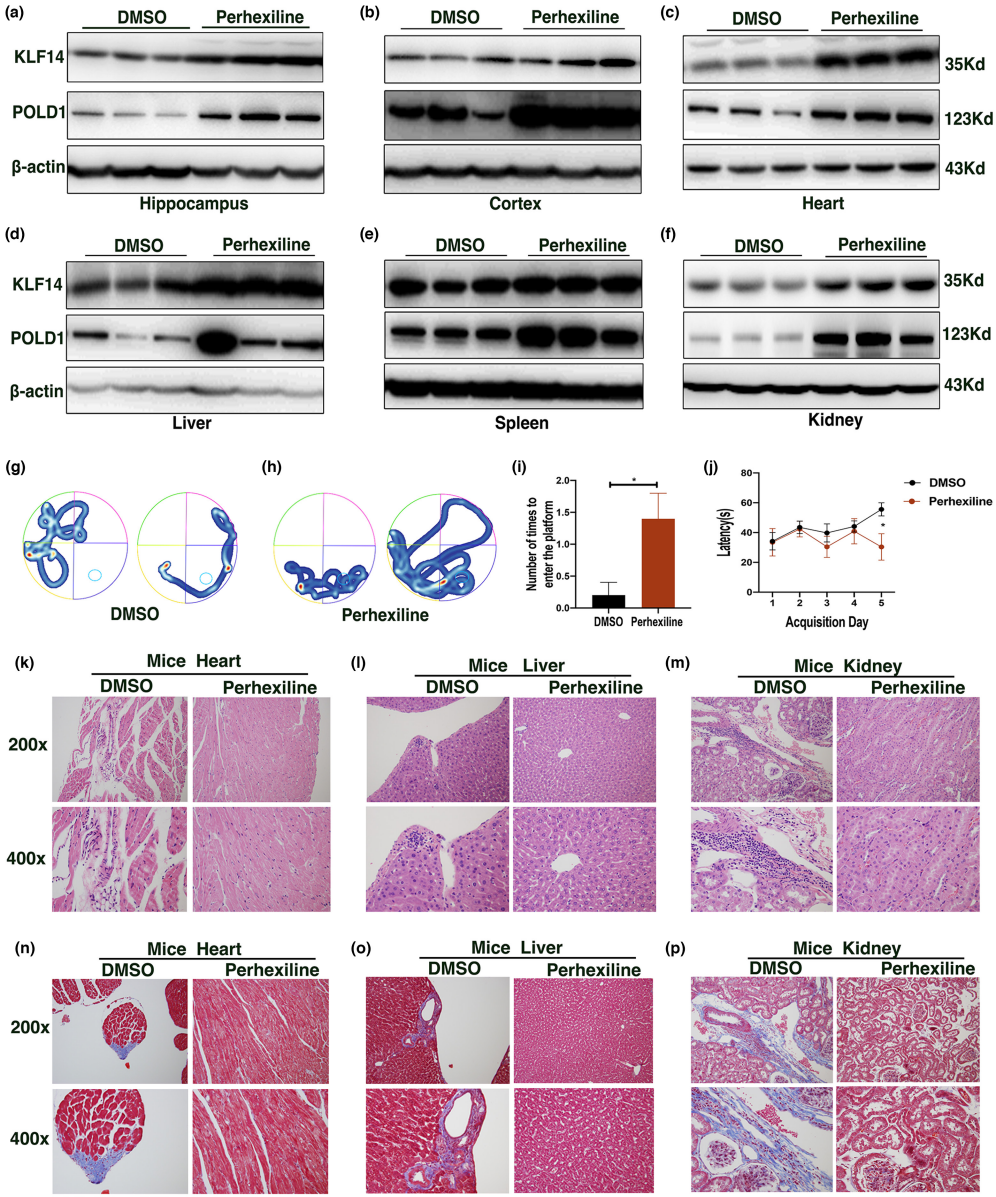
Perhexiline rescued aging phenotypes in senescence‐accelerated P8 (SAMP8) mice. (a–f) Krüppel‐like factor 14 and POLD1 protein expression levels in the hippocampus (a), cortex (b), heart (c), liver (d), spleen (e), and kidney (f) of 6‐month‐old SAMP8 mice (n = 6 per group) treated with DMSO or perhexiline (10 mg/kg) were determined by western blot. (g, h) Morris water maze (MWM) test representative trajectories of SAMP8 mice treated with DMSO (g) or perhexiline (h) in the probe trial. (i) The number of times the SAMP8 mice treated with DMSO or perhexiline entered the platform during the MWM test. (j) Latency (s) to find the platform of SAMP8 mice treated with DMSO or perhexiline during the MWM test. (k–m) Representative H&E staining images of the heart (k), liver (l), and kidney (m) of SAMP8 mice treated with DMSO or perhexiline. (n–p) Representative Masson trichrome staining images of the heart (n), liver (o), and kidney (p) of SAMP8 mice treated with DMSO or perhexiline. Data were compared by Student's *t* test, and data were shown as mean ± SEM (*p < 0.05).

Six‐month‐old SAMP8 mice were treated with 10 mg/kg body weight perhexiline (every other day for 6 weeks) to investigate whether KLF14 activation could delay aging. The 6‐month‐old SAMP8 mice were administered with a vehicle (DMSO) as control. We found that the expression level of KLF14 and POLD1 were upregulated in the hippocampus, cortex, heart, liver, spleen, and kidney tissues of the perhexiline‐treated mice compared with that in the control mice (Figure 7a–f, Figure S6a–l). Next, we detected the potential role of perhexiline on cognitive function by using a Morris water maze (MWM) test. During the probe trial in the hidden platform task, perhexiline‐treated mice crossed more times to reach the target platform compared with the control mice (Figure 7g–i). In addition, the results of the latency to the hidden platform showed that the perhexiline‐treated mice spent less time than the control mice (Figure 7j). Collectively, these data indicated that perhexiline strongly ameliorated spatial learning and memory impairment of SAMP8 mice.

Furthermore, a pathological examination of perhexiline‐treated mice tissues, including the heart, liver, and kidney, was performed. During gross dissection, control mice showed evident structural aberrations in viable organs such as the gastrointestinal tract, liver, kidney, and spleen compared with the perhexiline‐treated mice (Figure S6m,n). Histopathological examination of the vital organs revealed an abnormal pathological lesion in the control mice compared with the perhexiline‐treated mice using H&E staining and Masson's trichrome staining. The heart presented myofibrillar degeneration and collagen deposition, which were visualized as light blue staining, in the control mice compared with the perhexiline‐treated mice (Figure 7k,n, Figure S6o,r). In the liver, hepatocellular damage and inflammatory infiltrations were evident, and collagen deposition was observed in the control mice rather than in the perhexiline‐treated mice (Figure 7i,o, Figure S6p,s). The kidney exhibited degeneration in renal epithelial cells, inflammatory infiltrations, and fibrotic lesions in the control mice, and perhexiline could effectively alleviate kidney lesions in aged SAMP8 mice (Figure 7m,p, Figure S6q,t). These results demonstrated that perhexiline could lessen the development of age‐related tissue impairment and delay aging.

## 3 DISCUSSION

Considering the importance of cellular senescence in aging‐associated diseases, molecular mechanisms of cellular senescence require more detailed studies. In our research, we reported a key role of KLF14 in delaying cellular senescence and aging by promoting POLD1 transcription. In addition, the pharmacological activation of KLF14 confers protection against cellular senescence and aging‐related pathologies, supporting the therapeutic antiaging potential of KLF14 agonists.

Previous studies showed that KLF14 participates in several aging‐related diseases. Several lines of evidence suggest that KLF14 is a protective gene against atherosclerosis and diabetes via mediating lipid signaling and glucose metabolism (Chen et al., 2020). In addition, the reduction of KLF14 was reported to be related to abnormal centrosomal amplification and aneuploidy (Fan et al., 2015). Moreover, KLF14 could transactivate PPARγ promoter activity to ameliorate liver fibrosis, of which PPARγ played an important role in regulating cell proliferation and cell senescence (Jiang et al., 2022). Notably, the methylation status of the CpG island located in KLF14 was associated with aging in human blood, buccal cells, and bone samples, and it served as an age‐related epigenetic biomarker (Pan et al., 2020; Schwender et al., 2021; Woźniak et al., 2021). The hypermethylation of KLF14 could result in the dysregulation of the cell cycle and DNA repair ability by influencing the TRIM59 methylation status in patients with AD (Duan et al., 2022; Wezyk et al., 2018). Furthermore, the hypermethylation level of KLF14 was correlated to its downregulation in breast cancer cells (Chu et al., 2022). The above mentioned findings indicated a possible close relationship between KLF14 and aging.

A reduced KLF14 expression was reported in the adipose tissue of aged mice (Iwaya et al., 2018). Similarly, in our experiments, we found that KLF14 expression decreased with cellular senescence and aging, and the reduced KLF14 level was associated with a decline in cell proliferation, DNA synthesis, and DNA damage repair ability. Luo et al. (2019) and Boot et al. (2016) also showed that the overexpression of KLF14 could promote cell proliferation and cell cycle and decrease cell apoptosis in LNCaP cells and thyroid cancer cells. However, several studies reported that KLF14 overexpression inhibited cell growth and proliferation in breast cancer (Chu et al., 2022), colorectal cancer (Li et al., 2020), and KRAS oncogenic mutant cancer cells (Fernandez‐Zapico et al., 2011). This discrepancy remains unclear.

We used the KLF14 ChIP‐seq data to explore the functional enrichment of target genes, which enriched the KLF14 binding consensus motif. The functional annotations were primarily enriched in cellular senescence and cell cycle, which is consistent with the KLF14 function in our research. Among the KLF14 ChIP‐seq targets related to cellular senescence, some genes have been studied, such as TGFβRII and p65. KLF14 could bind to the TGFβRII promoter to repress the activity of TGFβ signaling which is a potent inducer of cellular senescence (Truty et al., 2009). KLF14 also could inhibit the expression of p65 to respond to inflammation, a prominent feature of the senescence‐associated secretory phenotype (SASP) (Hu et al., 2018). These results suggested that KLF14 may exert its function in cellular senescence via multiple genes. Given the importance of POLD1 in senescence, we analyzed whether KLF14 had a functional impact on the expression of the POLD1.

POLD1, which provides the essential catalytic activities of Pol δ, plays an important role in DNA synthesis and maintenance of genomic integrity (Nicolas et al., 2016). The downregulation of POLD1 suppressed cell proliferation and genome instability in HEK‐293 cells, human fibroblast cells, and immune cells (Nichols‐Vinueza et al., 2021; Song et al., 2015). A growing body of studies have implicated the defect of POLD1 in the progression of aging and aging‐related diseases. For example, the impairment of POLD1 activity has been identified in developmental disorders such as MDPL and Werner syndrome which are both characterized by signs of premature aging affecting more than one tissue or organ (Cenni et al., 2018; Lessel et al., 2015). In addition, decreased POLD1 expression has been observed in senescent cells when compared with younger ones from human skin fibroblasts or lymphocytes (Takahashi et al., 2005), which is consistent with our results. Notably, changes in POLD1 levels were positively associated with KLF14 levels and positively regulated by KLF14 in aging.

Based on previous reports, several transcription factors have been found to bind with the POLD1 promoter to regulate its expression. For example, Sp1 and p53 can activate or inhibit the POLD1 transcription by binding with its promoter (Antoniali et al., 2015). Truty et al. (2009) demonstrated that KLF14 could compete with Sp1 in binding to the TGFβRII promoter because they both bind similar GC‐rich DNA sequences. In our study, KLF14 can activate the POLD1 transcription by binding to its promoter, resulting in increased POLD1 expression. Furthermore, the level of KLF14 binding to the POLD1 promoter decreased with aging, which could be the exact mechanism by which KLF14 regulates POLD1 in aging.

Subsequently, we performed a rescue assay to identify the role of POLD1 on the function of KLF14 in cellular senescence. When POLD1 was silenced in human fibroblast cells, the KLF14‐mediated decrease of the positive SA‐β‐Gal staining rate, the increase of cell proliferation, DNA synthesis, and DNA damage repair ability were nearly reversed. Therefore, the effects of KLF14 on cellular senescence were mediated via POLD1 regulation.

The biological role of KLF14 in suppressing aging was further determined and confirmed by the deletion of KLF14 in mice. Ample evidence shows that cognitive decline commonly occurs with aging and as an early marker of pathological aging such as AD (Zlatar et al., 2022). In our research, a significant cognitive deficit was observed in KLF14^−/−^ mice compared with WT mice, which is likely due to the low KLF14 and POLD1 levels in the hippocampus and cortex of KLF14^−/−^ mice. In addition, a previous study has implicated KLF14 in modulating gene expression in neurons, resulting in the reentry of the neuronal cell cycle and DNA damage responses under pathological conditions in AD, which may explain the protective role of KLF14 in cognitive decline (Wezyk et al., 2018). Moreover, tissue aging was assessed by H&E staining and Masson's trichrome staining in the heart, liver, and kidney. Aged tissue is characterized by increased fibrosis, elevated inflammatory infiltration, and accumulation of damage (Tian et al., 2020). We provided strong evidence of fibrotic lesions, inflammatory infiltration, and cell degeneration in the heart, liver, and kidney of KLF14^−/−^ mice, which is likely due to the low KLF14 and POLD1 levels in the tissues. Moreover, recently reported findings revealed that KLF14 exerted a protective role in cardiovascular disease and multiple chronic liver diseases by inhibiting inflammation and fibrosis, which is consistent with our results (Du et al., 2021). Furthermore, we found atrophy in the spleen of KLF14^−/−^ mice with low KLF14 and POLD1 expression compared with WT mice. These findings indicated that KLF14 deficiency, accompanied by the downregulation of POLD1 expression, promoted aging and aging‐related pathology. The timepoints (3‐, 6‐, and 11‐month‐old) in our study were specifically chosen to examine the ages at which the mice were most likely to exhibit phenotypic differences. However, aging is a dynamic and continuous process that occurs over time at different levels of organs, further investigations are needed to continuously observe the difference of aging phenotypes between the two groups of mice during aging.

Previous studies have found that perhexiline can reduce the formation of atherosclerosis through the activation of KLF14 expression, and this strategy is currently used in the clinical treatment of cardiovascular diseases (Guo et al., 2015). In addition, the activation of KLF14 by perhexiline conferred protection against sepsis in mice by inhibiting the inflammatory signaling pathway (Yuan et al., 2022). In our research, perhexiline, as a KLF14 agonist, enhanced cell proliferation, DNA synthesis, and DNA damage repair to inhibit cellular senescence by promoting the expression of POLD1. We also conducted in vivo experiments to verify the antiaging function of perhexiline in SAMP8 mice, which exhibited accelerated aging with a shortened life span and age‐related deteriorations in multiple tissues (Katayama et al., 2022). Notably, the treatment of SAMP8 mice with perhexiline improved cognitive function and reduced tissue lesions. Although the underlying molecular mechanism of the KLF14 agonist perhexiline in aging was complicated and was unclear in our study, we hypothesize that its antiaging function could be due to the activation of KLF14. Moreover, KLF14 could be a potential new target for delaying aging, and related clinical trials are warranted.

**FIGURE 8:**
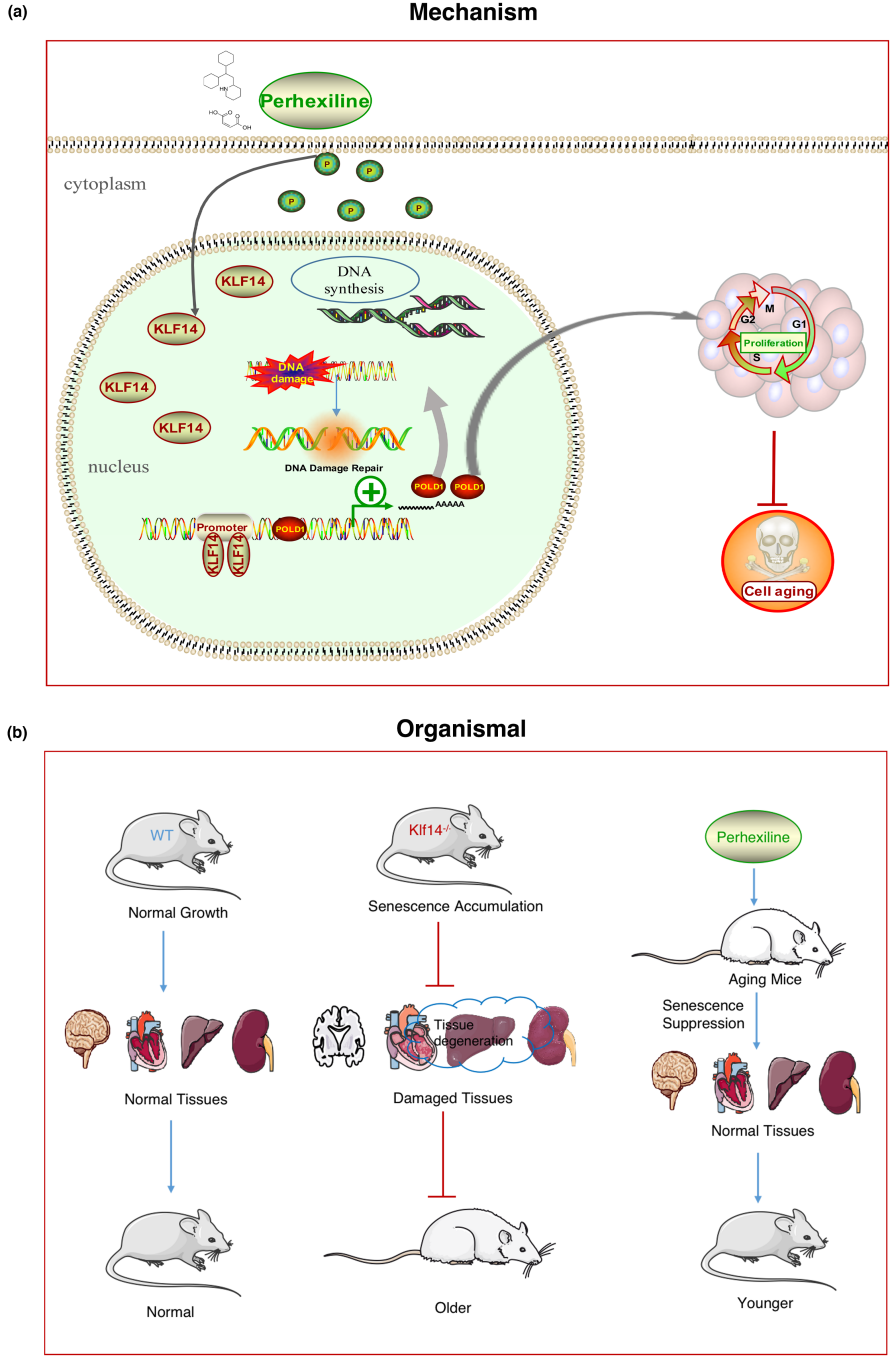
Diagram showing the mechanism by which KLF14 positively regulates POLD1 expression to suppress cellular senescence and aging. (a) The mechanism of KLF14 in cellular senescence. (b) The effect of KLF14 in mouse aging.

In this study, we identified KLF14 as a key suppressor of senescence and aging. KLF14 represses senescence and aging by promoting POLD1 expression in vitro or in vivo (Figure 8). KLF14 may play important roles in many biological processes apart from those investigated herein, which makes KLF14 and its agonists potential agents for the prevention of aging and aging‐associated diseases. The ChIP‐seq results indicated that KLF14 may bind to the promoter of other genes in addition to POLD1 to modulate aging progression, suggesting that there is likely greater complexity of this regulatory network, which is worthy of further study.

## 4 METHODS

### 4.1 Cell lines and culture conditions

Human embryonic lung diploid fibroblast cells (2BS cell line), human lung fibroblast cells (WI38 cell line), and human embryonic kidney 293 cells (HEK‐293) were obtained from the National Infrastructure of Cell Line Resource. HEK‐293 cells were cultured in Dulbecco's modified Eagle's medium (Gibco, Life Technologies) and supplemented with 10% fetal bovine serum (FBS; Gibco, Life Technologies) and 1% penicillin–streptomycin (Gibco, Life Technologies) at 37°C with 5% CO_2_. 2BS and WI38 cells were cultured in Minimum Essential Medium (Gibco, Life Technologies) with 10% FBS and 1% nonessential amino acids (Gibco, Life Technologies) and 1% penicillin–streptomycin at 37°C with 5% CO_2_. Cell cultures were expanded through sequential subculturing using trypsin–EDTA (Gibco, Life Technologies) to achieve a higher PD level. 2BS and WI38 cells were considered young at PD30 or below, and they became senescent when being passaged (Dang et al., 2020).

### 4.2 KLF14 knockout mouse generation

The KLF14^−/−^ mouse was generated using CRISPR/Cas9‐mediated gene editing system. Exon 1 of mouse KLF14 (Ensembl: ENSMUSG00000073209) was targeted with two guide RNAs (gRNA1: 5′‐CGCGATCCAGGGGTGTCCTCGGG‐3′, gRNA2: 5′‐GAATGGACAGAGAGAAGGCATGG‐3′) to introduce two DNA breaks with Cas9 endonuclease. gRNAs and Cas9 nuclease mRNA were transcribed in vitro and were injected into the fertilized eggs of C57BL/6J mouse and implanted into the surrogate mothers to obtain founders. The founders were identified by PCR genotyping and confirmed by DNA sequencing analysis. They were then bred with wild‐type C57BL/6J mouse to produce generation 1 (F1) which was confirmed by PCR genotyping and by DNA sequencing analysis. F1 mice were bred to establish mice colonies. The KLF14^−/−^ mouse model was generated with assistance from Shanghai Model Organisms Center, Inc. The following primer sequences were used to identify mouse genotypes: 5′‐AAAAAGCCCCCACAGTCATAAACC‐3′ and 5′‐CTGGGGCAATCGGGAGAGTCAAAG‐3′.

### 4.3 Animals

Senescence‐accelerated P8 (SAMP8) mice and senescence‐resistant R1 (SAMR1) mice were purchased from Beijing HFK Bioscience. They were divided into three groups in accordance with age: 2 months (2M, n = 6), 4 months (4M, n = 6), and 8 months (8M, n = 6). Senescence‐resistant R1 (SAMR1) and senescence‐accelerated P8 (SAMP8) mice were a suitable model for the study of aging, and SAMR1 animals can serve as SAMP8 controls (Flood et al., 1993). C57BL/6J WT mice were purchased from Beijing HFK Bioscience. Mice were housed in groups of five animals per cage in a temperature‐controlled facility and kept on a regular 12‐h light and dark cycle. Mice were maintained, and experimental procedures were performed under pathogen‐free conditions. All animal experiments were approved by the Bioethics Committee of Xuanwu Hospital of Capital Medical University and in compliance with the National Institute of Health Guide for the Care and Use of Laboratory Animals.

### 4.4 Perhexiline treatment

The cells were treated with DMSO (Sigma Aldrich) or perhexiline maleate salt (Sigma Aldrich, 2 and 5 μM), which was dissolved in DMSO for 24 h. The SAMP8 mice were treated three times a week (Monday, Wednesday, and Friday) with perhexiline (10 mg/kg) or DMSO for 6 weeks via gavage administration.

### 4.5 Actinomycin D treatment

Actinomycin D (ActD, Med Chem Express) was dissolved in DMSO. 2BS and WI38 cells were initially infected with lentivirus to overexpress KLF14 and then treated with 10 μg/mL of ActD or DMSO for 3 h to inhibit gene transcription. Treated cells were collected for RNA isolation and real‐time PCR analysis.

### 4.6 Western blot

Total cellular and tissue proteins were extracted by adding RIPA (Beijing Solarbio Science & Technology Co., Ltd.) buffer containing protease inhibitors (Beijing Solarbio Science & Technology Co., Ltd.). The protein concentration was determined using a BCA protein assay kit (Thermo Fisher Scientific). Equal amounts of protein were loaded for SDS‐PAGE electrophoresis. Then, the protein from gels was transferred to PVDF membranes (Millipore). The membranes were blocked by immersing in 5% nonfat milk in TBST for 1 h to inhibit nonspecific binding and then incubated with the primary antibodies KLF14 (1:1000, ABclonal), POLD1 (1:1000, Abcam), and β‐actin (1:5000, Zhongshan Boil Tech Co.) at 4°C overnight.

### 4.7 RNA isolation and RT‐qPCR

Total RNA from tissues and cells was purified using TRIZOL reagent (Thermo Fisher Scientific). cDNA was synthesized using a reverse transcription kit (Beijing Lablead Biotech Co., Ltd) following the manufacturer's protocol. Then, quantitative RT‐qPCR was performed using SYBR green reagents (Takara) on a Roche 480 machine. Primer pairs for RT‐qPCR are shown in Table 1. Gene expression was presented as a fold increase compared with RNA isolated from the control group by using the comparative CT (2^−ΔΔCT^) method.

**TABLE 1:** Primer sequences for RT‐qPCR and ChIP‐qPCR.

Gene	Primer sequences
Primers for real‐time PCR	
Human‐KLF14	F: 5′‐GGAACTGATAAGAAGGGATGAACT‐3′
	R: 5′‐CCTGGTGGATGGGTGAGACA‐3′
Human‐POLD1	F: 5′‐GCTCCGCTCCTACACGCTCAA‐3′
	R: 5′‐GGTCTGGTCGTTCCCATTCTGC‐3′
Human‐β‐actin	F: 5′‐ACAGAGCCTCGCCTTTGC‐3′
	R: 5′‐CCACCATCACGCCCTGG‐3′
Murine‐KLF14	F: 5′‐CTACAAGTCGTCGCACCTCAAGTC‐3′
	R: 5′‐CGCAGTCGAGCCAATCACAGG‐3′
Murine‐POLD1	F: 5′‐CGATGCCAACGCCAAGGTAGTC‐3′
	R: 5′‐CATTGCTTCAGCCACAGAGGAGAC‐3′
Murine‐β‐actin	F: 5′‐GGTCAGAAGGACTCCTATGTGG‐3′
	R: 5′‐TGTCGTCCCAGTTGGTAACA‐3′
Primers for ChIP‐qPCR	
Human‐POLD1 promoter site 1	F: 5′‐GCCTGTAGACCTATCGGCTCTCATC‐3′
	R: 5′‐GCGTGCTGGGCTTGAGGTGTAA‐3′
Human‐POLD1 promoter site 2	F: 5′‐CAGGGCCTTCCTCTTCCTCTGCTTT‐3′
	R: 5′‐TCAATCAGGTTCGGGCACCCAGT‐3′
Human‐POLD1 promoter site 3	F: 5′‐AACCATCCTCCTACCAAGACTCGGG‐3′
	R: 5′‐GGATTGGAGGAACTTCGTCGCTTCGC‐3′
Human‐POLD1 promoter site 4	F: 5′‐GGGACTTTAATGAGCGTGGCACTGT‐3′
	R: 5′‐CCGAACTCCTGGCGTCTCTCCTTT‐3′
Human‐POLD1 promoter site 5	F: 5′‐ACCGCACGAGGTCGTGAAGGTA‐3′
	R: 5′‐TCTCTCAGCTTCCGAAGTGGCCTC‐3′

Abbreviations: ChIP‐qPCR, chromatin immunoprecipitation‐qPCR; KLF14, Krüppel‐like factor 14; RT‐qPCR, reverse transcription PCR.

### 4.8 Human samples

Blood was drawn from the volunteers who signed informed consent. The volunteers included hospital staff, medical students, and healthy people recruited from the health screening center of Xuanwu hospital donors. The volunteers had no cancer, blood disease, and recent infection. In this study, participants were enrolled and divided into four age groups: ages between 20 and 29 years (n = 30, mean age of 25.3 years old), ages between 40 and 49 years (n = 15, mean age of 45.07 years old), ages between 60 and 69 years (n = 15, mean age of 65.13 years old), and ages between 80 and 89 years (n = 9, mean age of 84.78 years old). Peripheral blood mononuclear cells were obtained through Ficoll‐Paque (Beijing Solarbio Science & Technology Co., Ltd) density gradient centrifugation. The study was approved by the ethics board of Xuanwu Hospital Capital Medical University.

### 4.9 Chromatin immunoprecipitation (ChIP) assay

The ChIP assay was prepared in accordance with the procedure of the ChIP assay kit (Thermofisher). For the ChIP assay, 1.0 × 10^7^ cells, 10 μg of KLF14 antibody (DSHB), and 2 μL of normal rabbit IgG (Abcam) were used. DNA was collected from the processed tests utilizing PCR purification columns following the manufacturer's instructions (Qiagen). Then, real‐time PCR was used to detect the DNA sample and check the binding level.

### 4.10 Lentiviral transfection

In investigating the function of KLF14, cells were infected with lentiviral particles containing shRNA directed against KLF14 to knockdown KLF14 (shRNA‐KLF14) and scrambled control shRNA as negative control (shRNA). The overexpression of KLF14 was induced by lentiviral KLF14 (pLenti‐CMV‐KLF14) transfection and lentiviral packaging vectors as negative control (pLenti‐CMV). The stable POLD1 knockdown (shRNA‐POLD1) and overexpressed (pLenti‐CMV‐POLD1) cells were also established by lentiviral infection. All types of viruses used in this study were purchased from the Hanbio Biotechnology Co., Ltd. The supernatant was removed after 16 h and replaced with a fresh culture medium after the infection.

### 4.11 Senescence‐associated β‐galactosidase staining

Senescence‐associated β‐galactosidase (SA‐β‐Gal) staining was performed according to the manufacturer's instructions (Beyotime Biotechnology). Samples were incubated overnight with a β‐gal detection reagent at 37°C and visualized using a regular light microscope at 100× magnification (Olympus).

### 4.12 Cell counting kit‐8 assay

2BS and WI38 viability were monitored using a CCK8 kit (Beyotime Biotechnology) following the producer's instructions. For CCK8 detection, 10 μL of CCK8 reagent was added to the culture medium 2 h before analysis. Cell proliferation was monitored at different times (1–5 days).

### 4.13 Ethynyl deoxyuridine (EdU) incorporation assay

The EdU (Beyotime Biotechnology) kit was used to detect the DNA synthesis rate according to the maker's instructions. Then, the absorbance values of all wells at 370 nm were determined using an enzyme‐labeling instrument (Thermo Fisher Scientific).

### 4.14 Comet assay

Comet assay (Trevigen) was used to examine 2BS and WI38 cells against oxidative DNA damage under different conditions. After cells were transfected with the virus for 72 h, 100 μm of H_2_O_2_ was added to induce DNA damage. Comet assay was performed as previously described (Hou et al., 2021).

### 4.15 Dual luciferase reporter assay

The plasmids were purchased from SyngenTech. In brief, we cloned a 2000‐bp region of the POLD1 promoter (WT) or binding sites being mutated promoter sequence and inserted the cloned fragment into the pGL4.1 firefly luciferase reporter vector (Promega). An empty pGL4.1‐promoter vector was transferred as a systemic control. HEK‐293 cells were seeded into wells of a 24‐well plate and transiently transfected with various plasmids when cells were at 70% confluence. After 2 days, the luciferase activities were evaluated using the Lucifer Reporter Assay (Beyotime Biotechnology) and normalized to Renilla luciferase activity.

### 4.16 H&E staining

For histopathological examination, multiple tissues were dissected, fixed in 10% buffered formalin, and embedded in paraffin. Tissue sections were stained with H&E in accordance with the standard protocol (Beijing Solarbio Science & Technology Co., Ltd.). Multiple segments of each organ were analyzed by examiners who were blinded to the grouping of each sample. A light microscope equipped with a digital camera (COOLPIX 950, Nikon) was used for digitizing the histological images.

### 4.17 Masson trichrome staining

Masson trichrome staining was performed according to the manufacturer's instructions (Beijing Solarbio Science & Technology Co., Ltd.) to identify the fibrotic tissue. Multiple segments of each organ were analyzed by examiners who were blinded to the grouping of each sample. The images were digitized using a light microscope equipped with a digital camera (COOLPIX 950, Nikon).

### 4.18 Morris water maze

The MWM test was conducted to assess spatial learning and memory. The maze was filled with water and divided into four quadrants, with a platform placed in one quadrant. The MWM test included four platform trials per day for 4 consecutive days and a probe trial on the 5th day. In platform trials, each mouse was allowed to find the platform in 60 s. In the probe trial, the platform was removed, and each mouse was given 60 s to locate where the platform was originally placed. For these tests, the percentage of time in the target quadrant and target site crossings within 60 s were recorded. The time spent in the target quadrant was taken to indicate the degree of memory consolidation that had occurred after learning. All trials were monitored by using a video camera set above the center of the pool and connected to a video tracking system (Labmaze V3.0; Zhongshi Technology).

### 4.19 Bioinformatic analysis

The KLF14 ChIP‐seq dataset‐GSM3635904 was downloaded from a previous publication in NCBI GEO (https://www.ncbi.nlm.nih.gov/geo/). The GSM3635904 was the ChIP‐seq data from the homo sapiens HEK‐293 cell line using an eGFP‐KLF14 antibody. The Cistrome‐GO (http://go.cistrome.org/) webserver was used to perform functional enrichment analysis of KLF14 ChIP‐seq peaks of GSM3635904. Cistrome‐GO applies the minimum hypergeometric (mHG) test on the ranked gene list for each GO term and pathway. Moreover, mHG = 0.00898 and *p*‐value = 0.020 were used to analyze the GO/KEGG enrichment, and visualization was performed using “ggplot2” R packages.

### 4.20 Statistical analysis

The statistical examination was conducted using GraphPad Prism version 8 (GraphPad Software). All data were displayed as mean ± standard error of the mean (SEM). The difference between the two groups was analyzed using a two‐tailed *t* test with variance equality. Differences among the groups were analyzed by one‐way analysis of variance (ANOVA). The relationship between KLF14 and POLD1 expression was calculated by Spearman's rho test. *p* < 0.05 was considered a statistically significant result.

## AUTHOR CONTRIBUTIONS

Yuli Hou, Qiao Song, and Peichang Wang contributed to the design of the experiments. Yuli Hou, Yaqi Wang, Yuting Cui, Xiaomin Zhang, Min Cao, and Huanli Duan contributed to the conduct of experiments. Yuli Hou, Jing Liu, Jingjing Zhang, Xiaoling Wang, Congcong Liu, and Chi Zhang contributed to the reagents. Yuli Hou, Qiao Song, and Peichang Wang contributed to writing and editing the paper. Yuli Hou, Jingxuan Fu, and Peichang Wang provided the funding.

## ACKNOWLEDGEMENTS

This work was supported by grants from the State Key Program of the National Natural Science Foundation of China (Code: 82030064), National Natural Science Foundation of China (Code: 81871714, 81901406, 82102487), Beijing Sail Plan for Talents Development (Code: ZYLX202114), Beijing Key Clinical Specialty, HUIZHI Talent Leadership Development Program of Xuanwu Hospital (Code: HZ2021PYLJ023), 2021 National Natural Youth Cultivation Project of Xuanwu Hospital, Capital Medical University (Code: QNPY2021036), and Beijing Postdoctoral Research Foundation.

## CONFLICT OF INTEREST STATEMENT

None declared.

## Supporting information


Figures S1–S6

## Data Availability

The data that support the findings of this study are available from the corresponding author upon reasonable request.
