# Supplementary material for: Downregulation of Krüppel‐like factor 14 accelerated cellular senescence and aging
Source: Aging Cell. 2023 Aug 8;22(10):e13950. doi: 10.1111/acel.13950 (PMC10577553; doi:10.1111/acel.13950)
Supplement: Supplementary file 1 — Figures S1–S6 [file ACEL-22-e13950-s001.docx]

**Supplementary**

**Fig. S1 KLF14 expression decreased with aging, and the downregulation of KLF14 accelerated cellular senescence.** (a–b) The protein (a) and mRNA (b) expression of KLF14 in the lymphocytes of healthy people of different ages. (c) The protein expression of KLF14 in the hippocampus of SAMR1 and SAMP8 mice. (d) The protein expression of KLF14 in the cortex of SAMR1 and SAMP8 mice. (e) The protein expression of KLF14 in the liver of SAMR1 and SAMP8 mice. (f–g) The protein (f) and mRNA (g) expression of KLF14 in 2BS cells at different PDs. (h–i) The protein (h) and mRNA (i) expression of KLF14 in WI38 cells at different PDs. (j–k) The protein (j) and mRNA (k) expression of KLF14 in 2BS cells transfected with shRNA-KLF14, pLenti-CMV-KLF14, or the control vector. (l–m) The protein (l) and mRNA (m) expression of KLF14 in WI38 cells transfected with shRNA-KLF14, pLenti-CMV-KLF14, or the control vector. Data were compared by one-way ANOVA and Student’s t-test, and data were shown as mean ± SEM, with three independent experiments in each group (**p* <0.05, ***p* < 0.01, ****p <* 0.005).

**Fig. S2 POLD1 expression was downregulated with aging and positively regulated by KLF14.** (a–b) The protein (a) and mRNA (b) expression of POLD1 in the lymphocytes of healthy people of different ages. (c) The protein expression of POLD1 in the hippocampus of SAMR1 and SAMP8 mice. (d) The protein expression of POLD1 in the cortex of SAMR1 and SAMP8 mice. (e) The protein expression of POLD1 in the liver of SAMR1 and SAMP8 mice. (f–g) The protein (f) and mRNA (g) expression of POLD1 in 2BS cells at different PDs. (h–i) The protein (h) and mRNA (i) expression of POLD1 in WI38 cells at different PDs. (j) The relationship between KLF14 (X-axis) and POLD1 (Y-axis) mRNA levels in lymphocytes of healthy adults, as well as 2BS and WI38 cells. (k–l) The protein (k) and mRNA (l) expression of POLD1 in 2BS cells transfected with KLF14 lentivirus. (m–n) Protein (m) and mRNA (n) expression of POLD1 in WI38 cells transfected with KLF14 lentivirus. (o–p) The mRNA expression of KLF14 in 2BS (o) and WI38 (p) cells transfected with pLenti-CMV-KLF14 or the control vector and treated with actinomycin D (ActD) or DMSO. Data were shown as mean ± SEM of three separate experiments (**p* <0.05, ***p* <0.01, ****p* <0.001). Statistical analyses were performed using one-way ANOVA, Student’s t-test, and linear regression analysis in Prism.

**Fig. S3 Silencing POLD1 blocked the KLF14-induced POLD1 expression.** (a–b) The protein expression of KLF14 (a) and POLD1 (b) in 2BS cells co-transfected with KLF14 and POLD1 lentivirus. (c–d) The protein expression of KLF14 (c) and POLD1 (d) in WI38 cells co-transfected with KLF14 and POLD1 lentivirus. (e–f) The mRNA expression of KLF14 in 2BS (e) and WI38 (f) cells co-transfected with KLF14 and POLD1 lentivirus. Data were compared using Student’s t-test, and data were shown as mean ± SEM, with three independent experiments in each group (ns *p* >0.05, **p* <0.05, ***p* < 0.01).


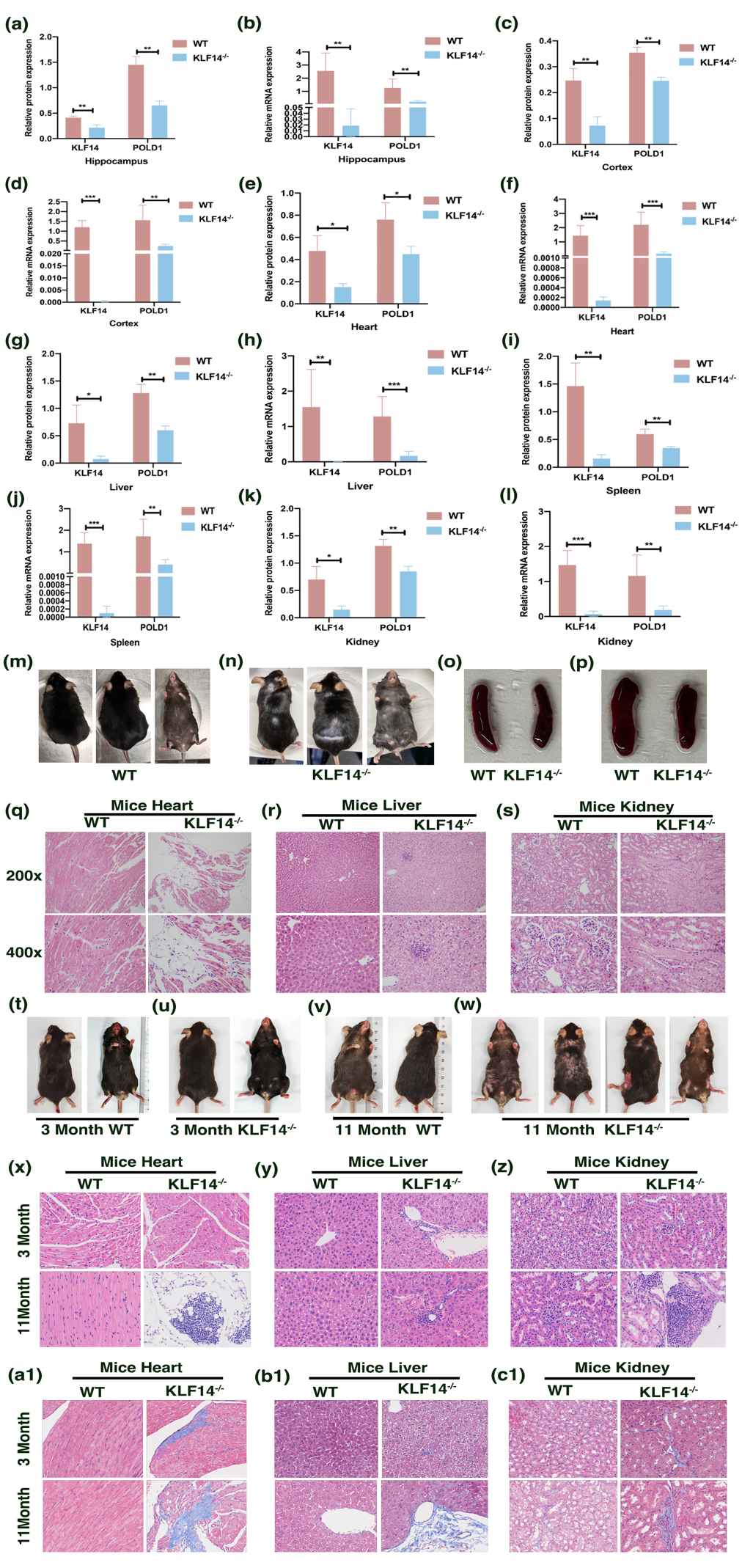


**Fig. S4 KLF14 deficiency promoted mouse aging.** (a–l) The protein and mRNA expression of the KLF14 and POLD1 in the hippocampus (a–b), cortex (c–d), heart (e–f), liver (g–h), spleen (i–j), and kidney (k–l) of 6-month-old WT and KLF14^−/−^ mice. (m–n) The fur images of 6-month-old WT and KLF14^−/−^ mice. (o–p) The spleen images of 6-month-old WT and KLF14^−/−^ mice. (q–s) Representative H&E staining images of the heart (q), liver (r), and kidney (s) of 6-month-old WT and KLF14^−/−^ mice. (t-u) The representative fur images of 3-month-old WT and KLF14^−/−^ mice. (v-w) The representative fur images of 11-month-old WT and KLF14^-/-^ mice. (x-z) Representative H&E staining images of the heart (x), liver (y), and kidney (z) of 3-month-old/11-month-old WT and KLF14^−/−^ mice. (a1-c1) Representative Masson trichrome staining images of the heart (a1), liver (b1), and kidney (c1) in 3-month-old/11-month-old WT and KLF14^-/-^ mice.

Data were compared by Student’s t-test, and data were shown as mean ± SEM (**p* <0.05, ***p* < 0.01, ****p* <0.001).

**Fig. S5 Perhexiline promoted POLD1 expression by activating KLF14.** (a–d) The protein and mRNA expression levels of KLF14 and POLD1 in 2BS (a–b) and WI38 (c–d) cells treated with DMSO or perhexiline. (e–h) The protein and mRNA expression of KLF14 and POLD1 in 2BS (e–f) and WI38 (g–h) cells transfected with shRNA-KLF14 or the control vector incubated with 5μM perhexiline. Data were compared using one-way ANOVA and Student’s t-test, and data were shown as mean ± SEM (**p* <0.05, ***p* < 0.01, ****p* < 0.005).


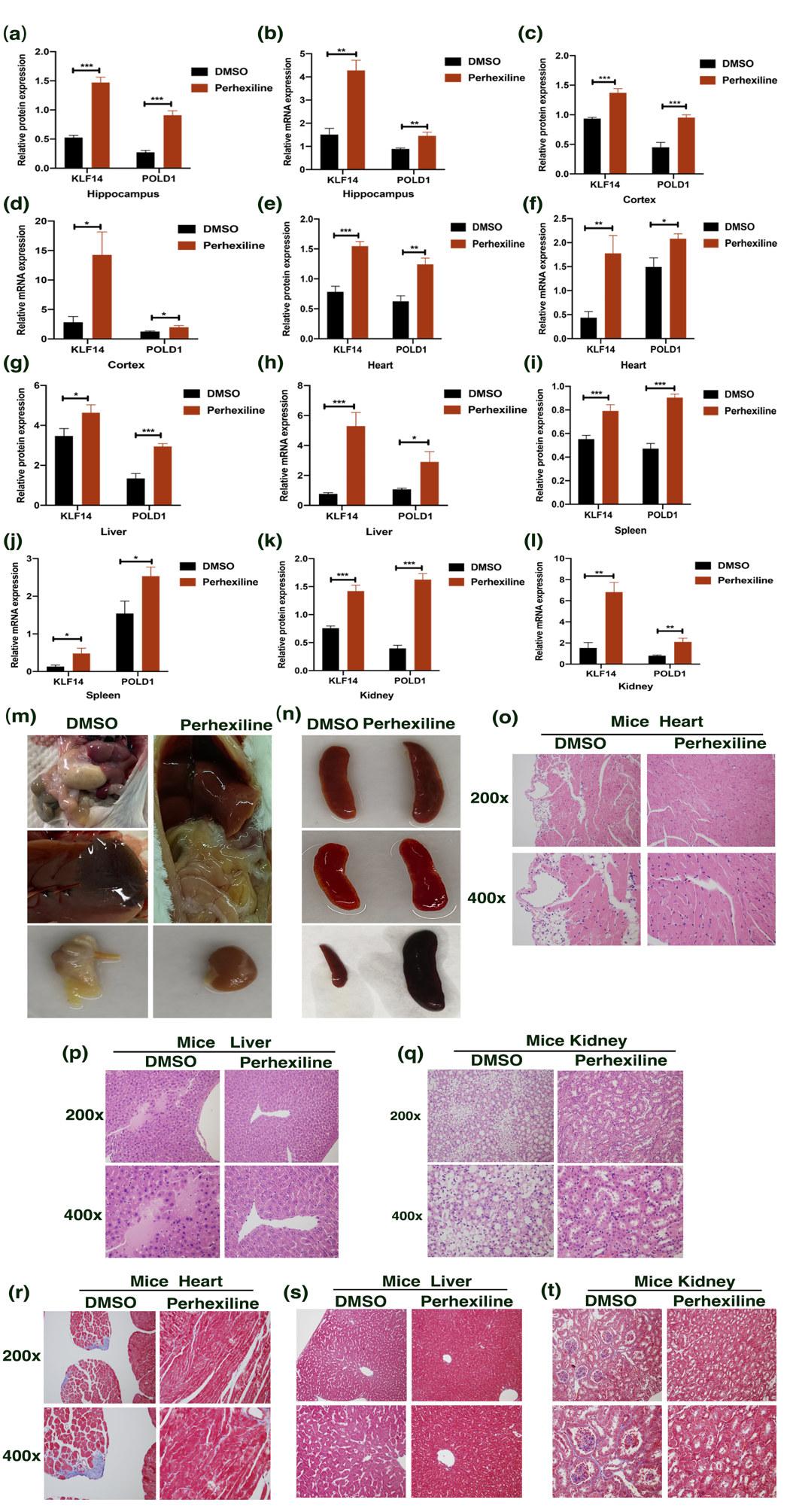


**Fig. S6 Perhexiline rescued aging phenotypes in SAMP8 mice.** (a–l) The protein and mRNA expression of KLF14 and POLD1 in the hippocampus (a–b), cortex (c–d), heart (e–f), liver (g–h), spleen (i–j), and kidney (k–l) of 6-month-old SAMP8 mice (n=6 per group) treated with DMSO or perhexiline (10 mg/kg). (m) The gross dissection images of SAMP8 mice treated with DMSO or perhexiline (10 mg/kg). (n) The spleen images of SAMP8 mice treated with DMSO or perhexiline (10mg/kg). (o–q) Representative H&E staining images of the heart (o), liver (p), and kidney (q) in SAMP8 mice treated with DMSO or perhexiline (10 mg/kg). (r–t) Representative Masson trichrome staining images of the heart (r), liver (s), and kidney (t) in SAMP8 mice treated with DMSO or perhexiline (10 mg/kg). Data were compared using Student’s t-test, and data were shown as mean ± SEM (**p* <0.05, ***p* < 0.01, ****p* <0.001).
